# Retraction: The Inhibitory Effect of Doxycycline on Cisplatin-Sensitive and -Resistant Epithelial Ovarian Cancer

**DOI:** 10.1371/journal.pone.0231890

**Published:** 2020-04-10

**Authors:** Wei Wu, Li-hua Yu, Bei Ma, Ming-juan Xu, The PLOS ONE Editors

After this article [1] was published, concerns were raised about results reported in Figs 2, 5 and 6.

Specifically:

- In Fig 2D, the lower portion of panel a appears similar to the upper portion of panel b. The authors reviewed the original data for this experiment and confirmed that the images in these panels were taken from the same culture plate.
- The ERK panels appear similar in Fig 5B and 5D, and in Fig 5F and 5H. In each case, the similar panels represent different experiments.
- Lanes 5, 6 of the Akt panel in Fig 5G appear similar to lanes 1, 2 of the p-Akt panel in Fig 6A, when the aspect ratio is adjusted.
- The p-ERK panels appear similar in lanes 1, 2 of Figs 5D and 6B, when the aspect ratio is adjusted.
- The β-actin panels for Figs 5A, 5C, 5E, 5G, 6A and 6C appear to be duplicated in Figs 5B, 5D, 5F, 5H, 6B and 6D, respectively. The similar β-actin panels represent the same experimental conditions for each pair. The authors confirmed that for each experiment the same transfer membrane was used to obtain the corresponding p-Akt, Akt, p-ERK, and ERK data, and so the same β-actin data applied.

The authors noted that the original data are no longer available for the panels mentioned in the second, third, and fourth bullet points, and so these issues could not be clarified. The figure concerns outlined above also have potential implications for the reliability of quantitative data reported in Figs 2D, 5 and 6.

In light of the unresolved image issues and concerns about the reliability of the reported results, the authors and the *PLOS ONE* Editors retract the article.

The authors apologize for the issues with this article.
